# For Earache

**Published:** 1886-11

**Authors:** 


For Earache.—Moure, in the *Revu d' Otologie*, etc., gives the following mixture:

| | | |
|---|---|---|
| ℞ | Sulphate of Atropia, - - - . | 1 part. |
| | Muriate of Morphia, - - - - | 2 " |
| | Neutral Glycerin, - - | 150 " |

A few drops to be introduced into the external auditory canal.—

